# Association Between Blood Cadmium Levels and Mortality in Peritoneal Dialysis

**DOI:** 10.1097/MD.0000000000003717

**Published:** 2016-05-13

**Authors:** Cheng-Chia Lee, Cheng-Hao Weng, Wen-Hung Huang, Tzung-Hai Yen, Ja-Liang Lin, Dan-Tzu Lin-Tan, Kuan-Hsing Chen, Ching-Wei Hsu

**Affiliations:** From the Department of Nephrology and Division of Clinical Toxicology (C-CL, C-HW, W-HH, T-HY, J-LL, D-LT, K-HC, C-WH), Chang Gung Memorial Hospital, Taipei; Department of Nephrology and Division of Clinical Toxicology (C-CL, C-HW, W-HH, T-HY, J-LL, D-LT, K-HC, C-WH), Lin-Kou Medical Center, Taoyuan; and Chang Gung University and School of Medicine (C-CL, C-HW, W-HH, T-HY, J-LL, D-LT, K-HC, C-WH), Taipei, Taiwan, ROC.

## Abstract

The negative impact of environmental exposure of cadmium has been well established in the general population. However, the effect of cadmium exposure in chronic peritoneal dialysis (PD) patients remains uncertain.

A total of 306 chronic PD patients were included in this 36-month observational study. Patients were stratified into 3 groups by the tertile of baseline blood cadmium levels (BCLs): high (>0.244 μg/L, n = 101), middle (0.130–0.244 μg/L, n = 102), and low (<0.130 μg/L, n = 103) for cross-sectional analyses. Mortality rates and cause of death were recorded for longitudinal analyses.

Patients in the high-BCL group were older, more likely to have diabetes mellitus, had lower levels of serum albumin and lower percentage of lean body mass than patients in the low-BCL group. A multivariate logistic regression analysis revealed that logarithmic transformed BCL was independently associated with a higher risk of low turnover bone disease (odds ratio = 3.8, *P* = 0.005). At the end of the 36-month follow-up, 66 (21.6%) patients died. Mortality rates increased with higher BCLs (*P* for trend = 0.005). A Cox multivariate analysis showed that, using the low-BCL group as the reference, the high-BCL group had increased hazard ratios (HR) for all-cause mortality in chronic PD patients after adjusting for related variables (HR = 2.469, 95% confidence interval = 1.078–5.650, *P* = 0.043).

In conclusion, BCL showed significant association with malnutrition and low turnover bone disease in chronic PD patients. Furthermore, BCL is an important determinant of mortality. Our findings suggest that avoiding environmental exposure to cadmium as much as possible is warranted in chronic PD patients.

## INTRODUCTION

Cadmium is a toxic heavy metal that is known to cause health problems such as renal dysfunction and osteoporosis/osteomalacia in humans.^1–3^ The risk of all-cause and cardiovascular mortality was shown to be increasingly proportional to the degree of cadmium exposure in Japanese populations living in a cadmium-polluted area over 22 years of follow-up.^4,5^ Recently published data from 2 large-scale longitudinal studies from the United States and Sweden also suggested that environmental exposure to low levels of cadmium remains a critical determinant of all-cause and cardiovascular mortality.^6,7^ In addition, the association between cadmium and mortality persisted even after extensive adjustment for smoking status and traditional cardiovascular risk factors. The exact mechanism(s) underlying this association is far from clear, but may include nephrotoxicity, enhanced atherosclerosis, direct cardiac toxicity, and an increase in the formation of malignant neoplasms.^1,3,8,9^

Increased morbidity and mortality have been observed in individuals with end-stage renal disease (ESRD) compared with the general population.^10^ Patients with ESRD exhibit cadmium accumulation in bone tissue and have increased blood cadmium levels (BCLs).^11–13^ We recently reported that increased BCLs were independently associated with all-cause mortality in hemodialysis (HD) patients after 3 years of follow-up.^14^ Our data supported the hypothesis that this association might be partly mediated by malnutrition and chronic inflammation.^15^ Early identification of this vulnerable patient population and efforts to reduce further cadmium exposure could therefore be an important means of reducing mortality rates. However, the role of raised BCLs on long-term mortality in chronic peritoneal dialysis (PD) patients has not been elucidated. Solute removal in intermittent HD is periodic, and it is conceivable that BCLs in HD patients might exhibit interdialytic oscillation or a sawtooth pattern.^16^ In contrast, as chronic PD is a form of renal replacement therapy in which a steady state is achieved, it may be a more suitable model than HD to study determinants or long-term outcome of high BCL in dialysis patients. Furthermore, it is important to note that the difference in dialysis modality may affect the metabolic pattern or comorbid conditions of dialysis patients, suggesting that the generalizability of previous studies from maintenance HD patients to PD populations may be questionable. We therefore conducted a 36-month investigation to evaluate the relationship between BCL and mortality in chronic PD patients.

## MATERIALS AND METHODS

### Study Population

This clinical study was carried out in accordance with the Declaration of Helsinki and was approved by the Institutional Review Board of Chang Gung Memorial Hospital, a tertiary referral center located in the northern part of Taiwan. All study patients were recruited from the Peritoneal Dialysis Center of Chang Gung Memorial Hospital. Patients who were ≥18 years of age and who underwent PD for >3 months were included in the study. The exclusion criteria were as follows: a history of malignancy, active infection, known drug abuse, and hospitalization or surgery within 3 months preceding the initial assessment. Individuals with a history of occupational exposure to heavy metals or metal intoxication, or who lived in metal-contaminated areas were also excluded. A total of 306 patients (113 men and 193 women) were included and represented 86% of the total PD population in our center.

### Data Collection and Definitions

Clinical characteristics recorded included age, gender, body mass index, duration of PD, the presence of diabetes mellitus (DM), and hypertension. The presence of comorbid conditions was determined after an in-depth review of medical records, including history and physical examination, progress notes, discharge summaries, consultations, and biopsy results. Patients with DM were defined as those with a history of DM diagnosed by a physician, or any 2 consecutive fasting glucose levels >126 mg/dL. The presence of cardiovascular disease, including cerebrovascular disease, coronary artery disease, congestive heart failure, and peripheral vascular disease, was recorded. Patients with hypertension were defined as those taking antihypertensive drugs regularly, or those with a blood pressure >140/90 mm Hg at least twice. History of smoking, alcohol consumption, and drug use was also recorded. Bone biopsy remains the gold standard for diagnosis of renal osteodystrophy. However, based on previous reports that levels of intact parathyroid hormone (iPTH) <100 pg/mL are fairly reliable for the screening of low turnover bone disease, ^17^ patients with low turnover bone disease in this study were defined as those having iPTH level <100 pg/mL.

### Measurement of Cadmium and Peritoneal Cadmium Excretion

To ensure that the chronic PD patients were not exposed to dialysate cadmium contamination and to assess cadmium excretion via peritoneal dialysis, at least 2 samples were collected in cadmium-free plastic bottles that contained dialysate from 2-L PD dialysate bags before and after 4 hours of dwell in 13 patients. Cadmium levels were measured as previously described.^18^ Briefly, 900 μL of modifier solution in deionized water and 100 μL of whole blood, or 100 μL of modifier solution and 900 μL dialysate were added to a 1.5-mL Eppendorf tube and immediately shaken. After overnight storage at 4°C, the tubes were warmed to room temperature and then whirl-mixed for 5 to 10 seconds. The mixed sample was transferred to graphite furnace sampler cups and measured by electro-thermal atomic absorption spectrometry (SpectrAA-220Z; Varian, Palo Alto, CA) with Zeeman's background correction and an L’vov platform. The coefficient of variation for cadmium measurements was ≤5.0%. External quality control was maintained via participation in the National Quality Control Program conducted by the Taiwan government. All enrolled patients were then stratified based on BCLs into the following 3 equally sized groups for statistical analysis: low (1st tertile) BCL (<0.130 μg/L), middle (2nd tertile) BCL (0.130–0.244 μg/L), and high (3rd tertile) BCL (>0.244 μg/L).

### Laboratory Analysis

Blood specimens were collected within a few days of clinical examination to minimize the effect of any acute event. Fasting laboratory values measured included hemoglobin levels, albumin, creatinine, calcium, phosphorus, uric acid, total cholesterol, triglyceride, ferritin, and iPTH levels. Serum calcium was corrected for serum albumin according to the following formula: corrected calcium (mg/dL) = serum calcium (mg/dL) + 0.8 × [4.0 − serum albumin (g/dL)]. Serum iPTH was determined using a chemiluminometric immunoassay (ADVIA Centaur iPTH; Siemens Medical Solutions Diagnostics, New York, NY) with a reference range of 7 to 53 pg/mL. All other markers were measured using an automatic analyzer according to standard laboratory methods.

### Indices of Dialysis Adequacy and Assessment of Residual Renal Function

The 24-hour urine and dialysate samples were obtained to calculate the renal creatinine clearance (Ccr) and PD membrane characteristics, including peritoneal Ccr, total weekly Ccr (normalized to body surface area), and whole body weekly urea clearance (total Kt/V urea). A peritoneal equilibration test using the 4-hour standard method was used to determine PD transport characteristics. Residual glomerular filtration rate (GFR) and weekly residual renal function (RRF) were calculated as the average of the 24-hour urine urea and creatinine clearance. Dialysis prescription aimed at obtaining a total *Kt*/*V* of at least 1.7 per week.

### Assessment of Nutrition Status

Dietary protein intake was estimated from the protein equivalent of total nitrogen appearance (PNA) using the Randerson equation: PNA (g/24 h) = 10.76 (0.69 urea nitrogen appearance + 1.46). Urea nitrogen appearance was determined from measured urea excretion in urine and dialysate. PNA was normalized to body weight (nPNA). Lean body mass (LBM) was measured by the creatinine kinetics method, according to the formula recommended by Forbes and Brunining.^19^ LBM was normalized by body weight (LBM%).

### Study Outcome

All patients were followed up for 36 months after the initial assessment. Patients who underwent kidney transplantation, or were transferred to HD were censored at the time of transfer to alternative renal replacement therapy. Every death during the follow-up period was reviewed and assigned an underlying cause by the attending physicians. Cardiovascular-related death was defined as follows: (1) fatal myocardial infarction verified by electrocardiography/enzymatic criteria and/or coronary angiogram; (2) life-threatening arrhythmia; (3) fatal stroke diagnosed by appropriate imaging studies and neurological criteria; (4) acute decompensated heart failure; (5) sudden death. For patients who died in the hospital, information on determining whether it was a cardiovascular- or infection-related death was obtained from hospital discharge diagnosis and death certificate records. In the case of an out-of-hospital death, family members were interviewed by telephone to fully ascertain the circumstances surrounding the death. The outcomes of this analysis were categorized as either infection-, cardiovascular-, or other-cause of death.

### Statistical Analysis

Continuous variables are expressed as mean ± standard deviation, and categorical variables are expressed as numbers or percentages of each item. The Kolmogorov–Smirnov test was used to determine the normal distribution for each variable. As serum iPTH, residual renal function, and BCL did not yield normal distribution, logarithmic conversion was conducted. The differences of clinical characteristics among the 3 study groups were analyzed using 1-way analysis of variance (ANOVA) for normally distributed continuous variables, the Kruskal–Wallis test for non-normally distributed continuous variables, and the chi-square test for categorical variables. The trend effect across the different BCL groups was tested by the trend test. The post-hoc multiple comparisons with Bonferroni test were used to determine which means were significantly different from each other. Multivariate logistic regression analysis was performed to evaluate the relationship between BCL and low turnover bone disease. Finally, using the Cox proportional hazard model, all significant variables that had *P* values <0.05 in the univariate analysis were included in the multivariate analysis with forward stepwise procedure to identify factors determining patient mortality. Statistical analyses were performed using SPSS (version 20.0). A *P* value of < 0.05 was considered statistically significant.

## RESULTS

### Study Population Characteristics

This study enrolled a total of 306 chronic PD patients with a mean age of 49.3 ± 13.5 years, 63% of whom were female. The mean duration of PD was 46.1 ± 34.2 months. The median (interquartile range) BCL value was 0.176 (0.108–0.311) μg/L. Patients were stratified into tertiles based on the BCL: low BCL (<0.130 μg/L, median BCL = 0.086 μg/L, n = 103), middle BCL (0.130–0.244 μg/L, median BCL = 0.177 μg/L, n = 102), and high BCL (>0.244 μg/L, median BCL = 0.395 μg/L, n = 101). The baseline characteristics and laboratory parameters in the 3 groups are described in Table 1. A significant linear trend appeared in age, diabetes mellitus, corrected calcium, phosphate, logarithmic-transformed (log) iPTH, albumin, and LBM%. Patients in the high-BCL group were older than patients in the other 2 groups. Compared with the patients in the low-BCL group, those in the high-BCL group were more likely to have DM. They also exhibited higher serum corrected calcium but lower phosphate and log iPTH levels than patients in the low-BCL group. In comparison with patients in the low-BCL group, patients in the high-BCL group displayed low serum albumin levels and low LBM%, indicating poorer nutritional status. Although statistically insignificant, there was a trend toward lower nPNA in the high-BCL group. There was no significant difference in gender, body mass index, PD duration, or prevalence of hypertension and cardiovascular disease between the different groups. Unexpectedly, the high-BCL group did not have a higher proportion of smokers. The groups did not differ significantly in terms of erythropoietin dose, hemoglobin, uric acid, and ferritin levels. There were also no significant differences in other cardiovascular risk factors such as total cholesterol and triglyceride levels between the 3 groups.

**TABLE 1:**
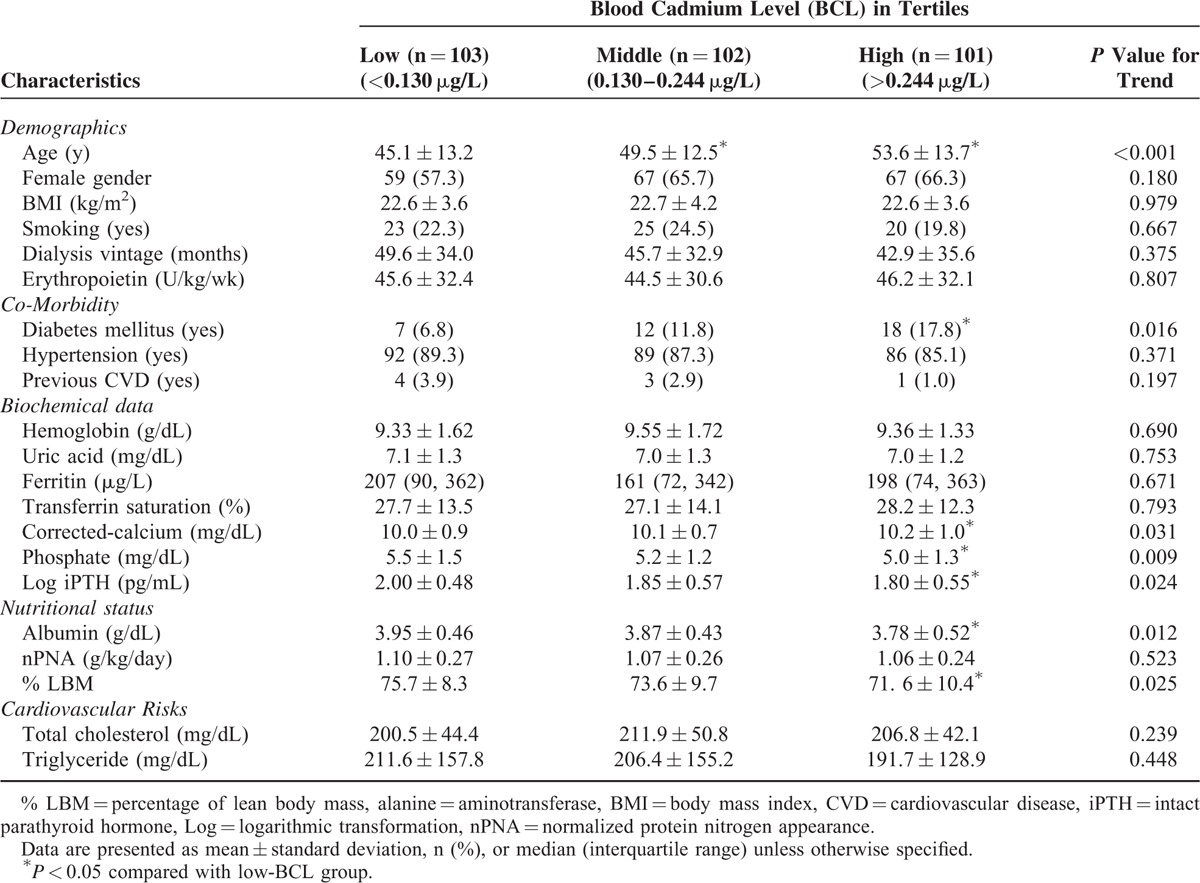
Comparison of Clinical Characteristics and Biochemical Data of Chronic Peritoneal Dialysis Patients Classified by Tertiles of Blood Cadmium Levels (n = 306)

### Baseline Peritoneal Membrane Characteristics, Dialysis Adequacy, and RRF

Baseline parameters of dialysis adequacy and peritoneal membrane characteristics in the 3 groups are described in Table 2. There was no significant difference in types of PD modality and transporter status between the groups. The mean total *Kt*/*V* urea of our patients was 2.17 ± 0.37, which meet the requirement of a minimal *Kt*/*V* urea target of 1.7. Overall, we did not observe a significant difference in peritoneal *Kt*/*V* urea, total *Kt*/*V* urea, peritoneal Ccr, and total weekly Ccr between the groups. There was no significant difference in the residual GFR or weekly RRF across the 3 tertiles of increasing BCL. There was also no significant difference in the number of patients with residual urine (defined as having daily urine >100 mL) between the 3 groups.

**TABLE 2:**
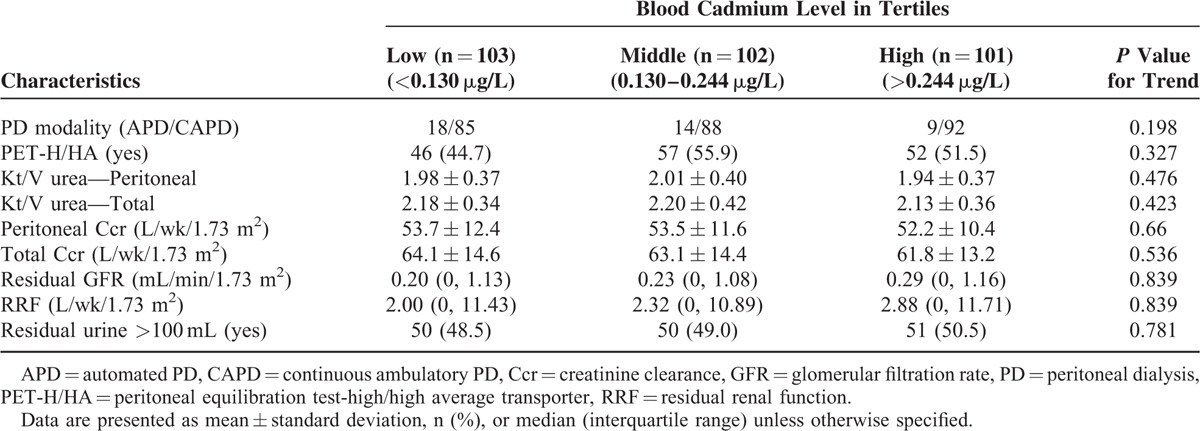
Comparison of Dialysis Adequacy and Peritoneal Dialysis-Related Data in Study Patients Classified by Tertiles of Blood Cadmium Levels (n = 306)

### Peritoneal Cadmium Excretion

The cadmium levels in the clear dialysate samples from the 13 chronic PD patients ranged from 0.01 to 0.19 μg/L. After 4 hours of abdominal dwell, the cadmium level in these dialysates ranged from 0.03 to 0.27 μg/L. With four 2-L exchanges per day, the calculated daily peritoneal cadmium excretion was 0.16 to 0.62 μg.

### Significant Correlation Between BCL and Low Turnover Bone Disease

**FIGURE 1:**
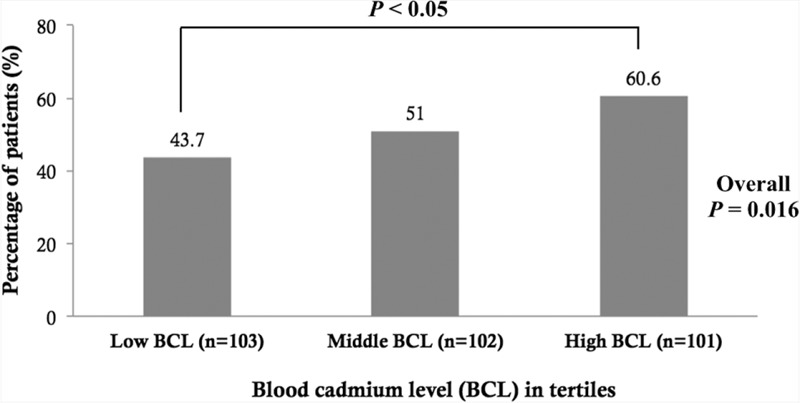
Percentage of patients with low turnover bone disease (defined by serum levels of intact parathyroid hormone < 100 pg/mL) in relation to the blood cadmium level in tertiles.

Of the 306 patients, 157 patients (51.3%) had low turnover bone disease. There was a significant increasing trend in the prevalence of low turnover bone disease according to BCL tertiles (43.7%, 51%, and 60.6%, respectively; *P* = 0.016) (Figure 1). Using multivariate logistic regression, the odds ratio and 95% confidence interval (CI) for low turnover bone disease were 3.8 (1.51–9.58) for each 1-unit of increased log BCL after adjustment for age, dialysis duration, serum albumin, residual renal function, and the presence of DM (Table 3, *P* = 0.005).

**TABLE 3:**
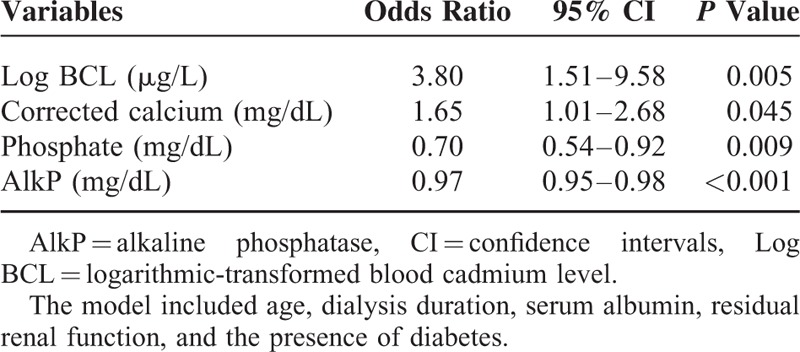
Multivariate Logistic Regression Analysis of Factors Associated With Low Turnover Bone Disease (n = 306)

### Multivariate Cox Regression Analysis of 36-month Mortality

There were a total of 66 (21.6%) deaths over a 36-month observation period. The major causes of death were cardiovascular disease (59.1%) and infections (37.9%). Two patients (3%) died of malignancies. Of the surviving study patients, 162 patients (52.9%) remained on PD treatment, 72 patients (23.5%) switched to hemodialysis, and 6 patients received renal transplantation. Of the 66 patients who died, 16 (15.5%) patients were in the low-BCL group, 18 (17.6%) patients were in the middle-BCL group, and 32 (31.7%) patients were in the high-BCL group (*P* for trend = 0.005). After adjusting for all significant factors, the multivariate Cox proportional hazard model showed that high BCL was associated with increased risk for all-cause mortality (adjusted hazard ratio = 2.469, 95% CI = 1.078–5.650, *P* = 0.043) compared with low BCL (Table 4). However, the BCL tertile was not a significant independent predictor of the 36-month cardiovascular- and infection-related mortality after adjusting for confounders.

**TABLE 4:**
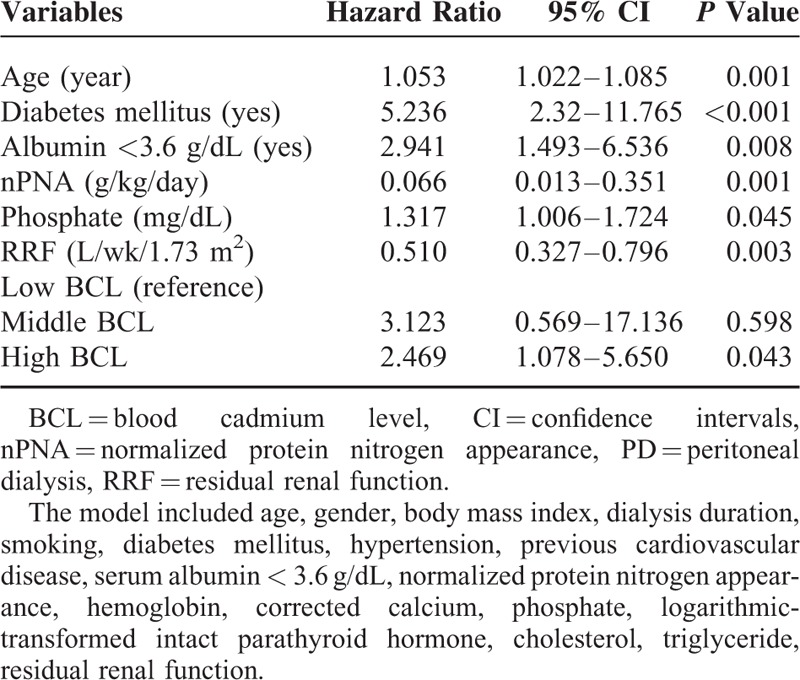
Multivariate Cox Regression Analysis of the All-Cause Mortality in Chronic Peritoneal Dialysis Patients, According to Baseline Prognostic Factors and Tertile of Blood Cadmium Levels (n = 306)

## DISCUSSION

The impact of environmental cadmium on health has been well established.^2,3,6,7^ The 1999–2004 National Health and Nutrition Examination Survey evaluated a cohort of nearly 9000 participants and showed that a higher quintile of BCL was independently associated with an ∼50% increase in all-cause mortality.^6^ Our previous study have attempted to address similar question in dialysis population. After taking relevant confounders into account, we demonstrated that BCL was associated with a higher risk of all-cause and cardiovascular-related mortality in 932 patients on maintenance HD.^14^ However, it was not clear if this relationship was true for chronic PD patients. Our present study showed that age, presence of DM, serum albumin<3.6 g/dL, and weekly RRF were all associated with an increased risk of all-cause mortality over 3 years of follow-up. Although these factors are well-established risk factors for mortality, we reported the novel finding that chronic PD patients with high BCLs (median BCL = 0.395 μg/L) had a significantly higher risk of all-cause mortality compared to patients with low BCLs (median BCL = 0.086 μg/L).

After uptake from the lung or gastrointestinal tract, there is a progressive accumulation of cadmium in the liver and kidney. Although cadmium is excreted mainly in urine, the amount of daily urine cadmium represents only a small fraction (∼0.004 %) of the total body burden, which leads to a long biological half-life of cadmium in the kidney cortex.^20^ Urinary cadmium is therefore considered a biomarker of cumulative body burden.^4,6^ Despite fluctuations due to recent exposure, BCLs are also influenced by the total body burden of cadmium and may serve as a good estimate of cumulative exposure in the general population.^1,6^ This is especially important for dialysis patients, the majority of whom have anuria. Compared with our previous cohort of patients on maintenance HD, the chronic PD patients in our present study had lower median BCL values (0.176 μg/L vs 0.367 μg/L). The relatively younger age (49.3 ± 13.5 years vs 56.1 ± 13.5 years) and shorter median duration of dialysis (3 years vs 6 years) in the present study may contribute to this difference. Several studies have reported that age is an important determinant of BCL.^3,21,22^ BCLs have also been shown to be positively correlated with dialysis duration.^13^ It is possible that most of the PD patients in our study had a higher RRF than our previous HD cohort, suggesting that higher BCL may be due to a decreased renal excretion. However, remnant renal excretion, analyzed as either a categorical variable (residual urine >100 mL) or a continuous variable (residual GFR), was not a critical determinant of BCL in our PD patients (data not shown). Strictly speaking, the degree of renal failure as residual GFR between 0 and 6.33 mL/min/1.73 m^2^ (the range of our present study) does not affect BCL. Furthermore, our study showed that the daily cadmium excretion in the peritoneal dialysate (0.16–0.62 μg) was similar to that in the urine of the general population (around 0.1–0.8 μg)^20^, suggesting the difficulty in removing cadmium from PD. Our data were consistent with this observation and showed no significant correlation between BCLs and any of the parameters of solute clearance in PD patients. Indeed, as plasma cadmium circulates mainly bound to albumin and metallothionein, it is plausible that the dialytic removal of these complexes via PD is limited because of their large molecular weights. Our study excluded chronic PD patients with high levels of exposure to cadmium, such as patients with occupational exposure or those from metal-contaminated areas. BCLs in our study therefore indicated chronic environmentally low-level of exposure. To our knowledge, our study is the first to show that high BCLs resulting exclusively from environmental exposure were associated with an increased risk of all-cause mortality in chronic PD patients.

There are several potential mechanisms to explain the association between cadmium and all-cause mortality in chronic PD patients. First, patients with high BCL exhibited a significantly lower serum albumin and LBM%. Albumin is predominantly synthesized by the liver and is downregulated during inflammation, whereas LBM is a reliable indicator of muscle mass and reflects somatic protein stores. The combination of these 2 factors suggested a malnutrition–inflammation complex syndrome in our chronic PD patients with high BCL, as previously described.^23^ Two large-scale population-based studies demonstrated a clear association between chronic cadmium exposure and systemic inflammation.^24,25^ These studies were consistent with our previous findings which showed that higher BCL was associated with an increased risk of inflammation as denoted by high-sensitivity C-reactive protein >3.0 mg/L.^15^ At the cellular level, cadmium induced oxidative stress as evidenced by the suppression of antioxidant defense systems, displacement of redox-active metals, and mitochondrial damage.^26^ Taken together, increased oxidative stress and chronic inflammation from toxic cadmium injury lead to malnutrition, increased rate of protein depletion from skeletal muscle, fragility, and an increased susceptibility to morbidity and mortality.

Second, the association between cadmium exposure and mortality can be explained by epidemiological studies, which suggested a link between cadmium exposure and the prevalence of either peripheral artery disease^27^ or atherosclerotic plaques in the carotid artery.^28^ The Malmö Diet and Cancer Study of 4819 participants followed for a total of 77 400 patient-years reported that the highest quartile of BCL was associated with a higher risk of major adverse cardiac events and cardiovascular-related mortality compared to the lowest quartile.^7^ Low-level cadmium exposure can cause vascular endothelial damage via oxidative stress and promote atherosclerotic plaque formation in ApoE knock-out mice.^8^ In our present study, cardiovascular-related mortality was the leading cause of death (39/66, 59.1%) over 3 years of follow-up. However, high BCLs did not have an independent effect on cardiovascular-related mortality. This could be due to the relatively small number of events, resulting in statistical tests that lacked power to detect this important association. In addition, our data showed that infection-related mortality was the second most common cause of death (25/66, 37.9%). Several studies showed an increase in cardiovascular events or mortality during hospitalization for acute infection.^29–31^ A study of 693 episodes of PD-related peritonitis reported that in 41.5% of patients with peritonitis-related mortality, the immediate cause of death was a cardiovascular event,^31^ supporting the idea that severe infection represents the final trigger in the pathological process leading to cardiovascular mortality among vulnerable individuals. The definition of some of these events as infection-related mortality in our present study could be another explanation for the lack of significance in cardiovascular outcome. Intriguingly, cadmium has been reported to cause immunotoxicity and decreased host defense in an animal model.^32^ It is therefore plausible that chronic cadmium exposure could result in a vicious cycle of chronic inflammation, atherosclerosis, and susceptibility to infection, leading to enhanced all-cause mortality in our chronic PD patients.

Finally, we speculate that there could be a potential link between cadmium-related bone disease and mortality in dialysis patients. Human and animal studies have demonstrated that the bone is a sensitive target of cadmium toxicity.^3^ Our present study illustrated a strong association between BCLs and low turnover bone disease as denoted by iPTH values lower than 100 pg/mL. Although bone biopsy remains the gold standard for diagnosis of renal osteodystrophy, iPTH values lower than 150 pg/mL have been shown to predict biopsy-proved low turnover bone disease in chronic PD patients with a specificity of 95.2% and a positive predictive value of 96.4%.^33^ Another analysis of bone biopsy results in 175 dialysis patients suggested that iPTH values lower than 100 pg/mL were fairly reliable for the screening of low turnover bone disease.^17^ Our data showed that the relationship between BCL and low turnover bone disease persisted even after adjusting for factors previously described as confounders.^34^ These data were in agreement with a previous study which showed a significant inverse association between serum PTH and BCLs.^35^ The mechanism underlying this association is thought to be mediated by increased osteoclast bone resorption, which mobilizes more calcium from skeletal to the circulation.^3^ Critically, accumulating evidence indicated that either low turnover bone disease or higher calcium load to the circulation may contribute substantially to increased death risk in dialysis patients.^34,36^ Moreover, low turnover bone disease in dialysis patients has been implied to be a consequence of the malnutrition–inflammation complex syndrome,^37^ providing further support to our first hypothesis.

Our study has several limitations. First, the cross-sectional nature of the data means that the temporal relationships between variables are not discernible, and our results indicate that they are hypothesis generating rather than hypothesis testing. Hence, correlations between the BCLs and increased mortality do not necessarily indicate causality. Second, the use of BCL as an exposure measurement might be another limitation of this study. Although toxicokinetic models were validated in general population, they are probably not appropriate to use among our chronic PD patients. However, our analyses showed that PD has very little effect on blood cadmium burden. Because of the cumulative property of cadmium, historically elevated exposures will still likely persist as elevated blood concentrations in our PD patients. Third, as the study was conducted at a single center with a smaller sample size compared to other national surveys, the potential of selection bias, and a type 2 statistical error could not be excluded. Moreover, as BCL measurement was performed at a single point of time, the amount of definite cadmium exposure over subsequent time periods could not be assessed. A future longitudinal prospective study with a larger sample size that measures cadmium exposure and BCLs at several points in time may be needed to evaluate the external validity of this study.

## CONCLUSION

Although our study cannot prove direct causality, this is the first study to demonstrate that high BCLs were associated with an increased risk for 36-month all-cause mortality in PD patients. Our findings suggest that high BCLs may have self-amplified adverse health effects in chronic PD patients. Further studies should aim to identify potential sources of cadmium exposure and to determine whether reduction of cadmium exposures provides a survival benefit in chronic PD patients.
